# Transcultural adaptation and psychometric study of the French version of the nursing home survey on patient safety culture questionnaire

**DOI:** 10.1186/s12913-019-4333-5

**Published:** 2019-07-15

**Authors:** Delphine Teigné, Guillaume Mabileau, Emmanuelle Anthoine, Marion Lucas, Brice Leclère, Leila Moret, Noémie Terrien

**Affiliations:** 1 QualiREL Santé, Hôpital Saint Jacques, 85 rue Saint Jacques, 44093 Nantes, France; 2 Public Health Department, University Hospital of Nantes, 85 rue Saint-Jacques, 44093 Nantes Cedex 1, France; 3 UMR INSERM U1246-SPHERE “methodS for Patients-centered outcomes & HEalth REsearch”, University of Nantes, University of Tours, 22 Boulevard Benoni Goullin, 44000 Nantes, France

**Keywords:** Nursing homes, Safety culture, Psychometrics, Survey, Risk management

## Abstract

**Background:**

The Nursing Home Survey on Patient Safety Culture (NHSOPS) questionnaire was developed by the Agency for Healthcare Research and Quality (AHRQ), particularly as an intervention to raise staff awareness about patient safety issues. The main objective of the present study was to provide a validated French-language measure of the safety culture (SC) in nursing homes. Thus the aim was i) to carry out a transcultural adaptation into French of the NHSOPS questionnaire, ii) to assess its psychometric properties in a sample of professionals working in French EHPAD facilities and iii) to develop our own tool.

**Methods:**

The study was carried out on volunteering professionals from 61 nursing homes (from January to March 2016). Two phases were conducted: an initial phase involving the translation and cultural adaptation of the questionnaire, and a second phase in which the psychometric properties of the questionnaire were assessed. A Structural Equation Model (SEM) with a maximum likelihood estimation method was used to evaluate the construct validity of the questionnaire. As the fit of the structure was not sufficient, an exploratory factor analysis using a principal axis factoring with an oblique rotation was then performed. Internal consistency was evaluated and we examined test-retest reliability using Intra-class Correlation Coefficients (ICC).

**Results:**

During the initial phase, all items were retained and minor adjustments were made. The participation rate by professionals was 58.4%. The exploratory analysis led to the identification of seven dimensions: Teamwork, Staffing, Compliance with procedures, Handoffs, Feedback and communication about incidents, Supervisor expectations and actions promoting resident safety, Overall perceptions of resident safety and Organizational learning. The SEM confirmed the existence of the seven latent dimensions (CFI = 0.946; TLI = 0.933; SRMR = 0.059; RMSEA = 0.061); internal consistency was acceptable. ICC per item ranged from 0.19 to 0.88.

**Conclusions:**

The results from this study were robust on seven dimensions. This French version is the first on Patient SC to have been applied to the medical-social sector caring for dependent elderly people in France. The NHSOPS questionnaire provides the opportunity to broach this subject. A national evaluation campaign should provide the opportunity to confirm or improve this measure.

**Trial registration:**

NCT02908373 (September 21, 2016) «Retrospectively registered».

**Electronic supplementary material:**

The online version of this article (10.1186/s12913-019-4333-5) contains supplementary material, which is available to authorized users.

## Background

In France, the number of dependent elderly people requiring daily care has been constantly on the rise in the last few decades [1]. As a consequence, accommodation for these people has seen many changes, particularly with the creation in France of accredited nursing homes for dependent elderly people (known as Etablissements d’Hébergement pour Personnes Agées Dépendantes – EHPAD). EHPADs are nursing homes that accommodate elderly people who are losing or have lost their physical and/or mental autonomy and cannot stay in their own homes [2]. These facilities provide assistance in daily living (getting up, going to bed, personal hygiene, meals etc) and services such as catering, laundry, and recreational activities. To complete this, medical and paramedical services are delivered by EHPAD salaried professionals (nurses and nursing assistants) and by professionals in private practice attached to the EHPAD (GP, physiotherapist, chiropodist etc). In France, over 585,000 elderly people were living in nursing homes in 2005 [3]. In a nursing home the living environment, where the concepts of home and institution are juxtaposed, is very different from the hospital care environment. Therefore any reflection needs to be adapted to the constraints and specificities of these nursing homes.

In France, as in other countries, care safety has been a public health concern for many decades in health facilities. It has arrived more recently in nursing homes. Improvement initiatives for care quality and safety in healthcare facilities generally integrate the promotion of the patient safety culture among professionals [4]. Although the efficacy of promotion of this sort in improving patient safety has rarely been studied [5–7], it is nevertheless a lever for the improvement of patient safety. It is supported by the 2012–2017 French national programme on the safety of healthcare. An assessment of healthcare teams’ safety culture enables scope for improvements to be identified [8], and, according to Shortell et al., it is one of the 4 main dimensions needed to reach objectives in terms of quality and risk management in healthcare facilities [9].

The safety culture (SC) in healthcare is a multidimensional concept for which there is no consensus definition, whether on the number, the nature or the definition of its different dimensions [10]. The European Society for Quality of Health Care defines SC as a coherent and integrated system of individual and organisational behaviours, based on shared beliefs and values, continuously striving to reduce damage caused to patients potentially linked to patient care procedures [11].

Among the existing questionnaires, the Hospital Survey on Patient Safety Culture (HSOSC) seems to be one of the most widely used questionnaires to assess the SC in health facilities. It was developed by the Agency for Healthcare Research and Quality (AHRQ) in 2004 [12] and has been translated and transculturally adapted many times throughout the world [13–16] as well as in France [17].

Several studies have shown the limitations of the HSOSC questionnaire in the context of nursing homes [18, 19]. This is why the AHRQ developed a similar questionnaire, adapted however to the specificities of nursing homes, namely the Nursing Home Survey on Patient Safety Culture (NHSOPS) questionnaire [20] which derives scores for 12 dimensions of SC.

This questionnaire is available in (Additional file 1). According to the AHRQ, the NHSOPS questionnaire can be used “as a diagnostic tool to assess the status of patient SC in a nursing home, as an intervention to raise staff awareness about patient safety issues, as a mechanism to evaluate the impact of patient safety culture improvement initiatives, and as a way to track changes in patient SC over time.” [21]. As in the original US version [21], to calculate subscale scores, the average percentage of positive responses on all items in each dimension is calculated. Dimensions are said to be “underdeveloped” if the score is <50%; “developed” if the score is > 75% and “developing” if the score is between 50 and 75% [21].

Many countries (Switzerland, Spain, Norway, Belgium, Germany and China) have translated, adapted and experimented on this questionnaire in the setting of their nursing homes [22–27]. The study of its psychometric properties has already been the basis of two European publications [25, 26], and one Asian publication [27].

The psychometric properties of the Swiss version of the NHSOPS scale were tested. Results of the factorial validity analysis showed nine dimensions, in contrast to 12 dimensions in the original NHSPSC scale [25]. According to these authors, the Swiss version needs further refinement and testing before its use can be recommended in Swiss nursing homes. The Norwegian version of the NHSOPS is fairly close to the Swiss version. The dimension analysis indicated that a modified ten-dimension model fitted the data-set in a Norwegian community healthcare context with acceptable goodness-of-fit values [24]. Moderate-to-strong correlations were found across the ten latent dimensions. The authors considered that this measure could be recommended as a useful tool to assess staff perceptions of patient safety issues in Norwegian nursing homes. Finally, a four-dimension structure was suggested after completing a principal axis dimension analysis for the Chinese version [27]. The researchers suggested caution in interpreting these findings, and considered that the stability of these item compositions should be tested in different Chinese populations in future studies.

The main objective of this study was to provide a validated tool in French language to measure the safety culture (SC) in nursing homes (EHPAD) Thus the aim was i) to perform a transcultural adaptation into French of the NHSOPS questionnaire, ii) to assess its psychometric properties in a sample of professionals in French EHPAD facilities, and iii) if it proved necessary to develop our own measure.

These reflections are part of a French research project on care system performances, called EPHAGE, coordinated by QualiREL Santé and funded by the Direction Général de l’Offre des Soins (DGOS) over the 2015–2017 period [28].

## Methods

### Questionnaire and variables

The NHSOPS is a self-administered questionnaire intended for all professionals working in EHPAD establishments. It comprises 42 items across 12 dimensions of the safety culture (Table 1), and comes under 4 headings: “work in your facility”, “communication”, “your hierarchy” and “your facility” (Table 1). The 42 items are scored on a 5-point Likert scale: (*never, rarely, sometimes, most of the time, always, or do not agree at all, do not agree, agree up to a point, agree, agree completely*). “Does not apply or don’t know” is included as a response category. Finally, the questionnaire includes items relating to socio-demographic characteristics of the professionals.

**Table 1:** The original patient safety culture dimensions of the NHSOPS used in the French nursing home study

Dimensions (*n* = 12)	Items
Teamwork	4
Staffing	4
Compliance with procedures	3
Training and skills	3
Non punitive response to mistakes	4
Handoffs (transfer of information)	4
Feedback and communication about incidents	4
Communication openness	3
Supervisor expectations and actions promoting resident safety	3
Overall perceptions of resident safety	3
Management support for resident safety	3
Organizational learning	4

### Study design

The adaptation and validation of the NHSOPS were conducted in a descriptive cross-sectional study. This was carried out in two phases: an initial phase in which the translation and cultural adaptation of the questionnaire was conducted and a second phase in which the psychometric validity was assessed.

### Phase 1: translation and transcultural adaptation

According to guidelines for a transcultural adaptation [29], the NHSOPS questionnaire was translated into French by two native French speakers with fluent English. One of the translators was acquainted with the concepts under assessment; the other translator was naive to the world of healthcare. Any divergence that stemmed from the translations was discussed by the project coordination team in presence of both translators. A first version of the questionnaire in French language was obtained. This first version was back-translated into English by two other bilingual translators whose mother tongue was English. They had previously neither taken part in the first translation work nor had any knowledge of the initial questionnaire. A meeting took place to harmonise the translations thus obtained with the 4 translators and the project coordination team, which yielded a second version of the questionnaire in September 2015.

The second version was tested to make sure that professionals understood the items and that the items were suited to the nursing home sector. Sixteen professionals (GPs, nurses, nursing assistants, catering staff, administrative staff, management) from 4 nursing homes were invited to fill in the questionnaire. They then were asked to evaluate it on the following criteria on an assessment grid: time taken to fill in the questionnaire, clarity of the documents, comprehension of the items and their appropriateness. These evaluations enabled a final version of the questionnaire to be reached.

### Phase 2: psychometric validation of the French version of the NHSOPS

#### Data collection and data management

The final version was used in a multi-centre study. The survey was carried out from January to March 2016 in 61 nursing homes volunteering to participate and different from those that had tested the questionnaire in Phase 1.

All salaried professionals working in the nursing homes, not only those directly involved in resident care, were invited to participate by a reference person in each facility. The reference person was a professional chosen by the management, and was in charge of the deployment of the process. Private practice professionals involved with the EHPADs were also included in the survey if their involvement in a nursing home was at least 10% of their working time. Indeed, the AHRQ recommends including private practice professionals in the survey even if they spend only a few hours a week in the nursing home [21]. The level of 10% of working time amounts to a half day of work in the week. This working time enables professionals to integrate into the organisation of the EHPAD and to interact with permanent salaried professionals, and thus to be sufficiently acquainted with the nursing home to respond to the survey.

The survey was conducted in four steps over a six-week period, as recommended in the AHRQ survey user’s guide [21]: 1) publicity and promotion of the survey; 2) questionnaire distribution; 3) issue of reminders; and 4) data collection closure.

All professionals received a questionnaire accompanied by a cover letter in which the purpose of the study was explained. They were asked to return the questionnaire anonymously to a drop-box in the facility. The responses to questionnaires were captured by an outside technician. The data capture was blind and in duplicate, with cross-checking. On reception in the database, the project coordination team members performed random checks of the data capture. Consistency between responses to the questionnaire by the professionals and the responses as captured in the database was checked on 40 questionnaires.

### Data analysis

The rate of participation by professionals were calculated in accordance with recommendations [21] according to the following calculation:

$$ participation\ rate=\frac{Number\ of\ surveys\ returned- incomplete\ }{Number\ of\ surveys\ distributed- ineligible\ } $$

The “number of surveys distributed” takes account of the surveys handed out by the reference person in the EHPAD; the “number of surveys returned” is the number of surveys posted into the drop-box; “incomplete surveys” are surveys that are returned in which no item is completed; respondents are considered to be “ineligible” when they were survey addressees but were not able to return the questionnaire in the time allowed (sick leaven end of contract, leave etc).

The statistical analyses used the usual techniques for descriptive statistics (frequency, means ± SD). The psychometric analyses were performed in several steps: the first step was to run a Structural Equation Model (SEM) using the maximum likelihood estimation method in order to confirm the structure of the questionnaire in 12 dimensions. This is a comprehensive statistical approach to test hypotheses about relationships among observed variables (i.e. items) and latent variables (i.e. dimensions) [30]. Four statistical indices were considered in order to verify model fit and to select the best-suited model: Steiger’s Root Mean Square Error of Approximation (RMSEA) and the Standardized Root Mean Square Residual (SRMR), with the fit considered as good when < 0.1, and very good when < 0.05; the Tucker Lewis Index (TLI) and the Comparative Fit Index (CFI) were considered as acceptable fit when > 0.9 [31]. Parameter estimations used the linear structural relationship approach developed by Jöreskog [32].

As these analyses revealed that the fit of the structure was not sufficient, an exploratory factor analysis (EFA) was then performed in order to identify the latent relational structure among items, leading to the identification of the new dimensions. Items with a percentage of non-available values (missing values and “Not Applicable” answers) of over 20%, or with a floor/ceiling effect over 50%, were eliminated. Missing data were not imputed, as they were missing at random. If two items exhibited a Spearman’s correlation coefficient over 0.8, only the more relevant of the two was kept (expert discussion). With the remaining items, the underlying dimensions were identified using a principal axis factoring on two thirds of the data randomly split, with a parallel analysis to determine the number of factors to retain [33]. This was also supported by the Kaiser-Meyer-Olkin (KMO) statistic for sampling adequacy, and the Bartlett’s test of sphericity. For an item to be attributed to a factor, the corresponding factor loading was to exceed 0.40. Eigenvalues and percentage of variance were also considered. An oblique rotation (oblimin) was used, as the factors identified were not totally uncorrelated (Additional file 2).

A final SEM was performed to examine the structure validity of the refined questionnaire on the remaining third of the data. The model hypothesized from the exploratory analysis was tested to confirm how well the data fitted the postulated structure.

Cronbach’s α-coefficients were computed to evaluate internal consistency [34]. Homogeneity was interpreted as acceptable if the alpha value was greater than 0.70 [35].

Finally, to evaluate reproducibility, a test-retest procedure was conducted in 5 nursing homes. Thirty-five professionals were asked to answer the questionnaire twice with a 2-week interval between test and retest. Intra-class Correlation Coefficients (ICC) were computed and considered satisfactory if greater than 0.60 [36].

All the study analyses were computed using R package version 3.4.3.

### Ethical and consent considerations

Participation was voluntary, and completion of the questionnaire was considered as informed consent. According to articles L1121–1 and R1121–2 in the French code of Public Health, Institutional Review Board (IRB) approval was not necessary.

## Results

### Translation and transcultural adaptation (phase 1)

All items were retained. Minor adjustments were made in the course of reconciliation meetings. They concerned choices of wording not affecting the patient safety subject matter and not specific to nursing home settings. For instance “this facility attaches importance to sharing the ideas and suggestions of staff members” versus “staff ideas and suggestions are valued in this nursing home” (item B7), or “ the management often goes round the home to assess the care given to the residents” versus “management often walks around the nursing home to check on resident care” (item D9).

All the professionals (*n* = 16) that were asked to make sure the items were comprehensible responded. Three quarters of the testing professionals responded positively to the statement: “I have all the necessary information to respond to the questionnaire”. The questions were clear for 93.8% of the respondents and 87.6% found the questionnaire easy to fill in. Completion of the questionnaire was timed at 16 min. The length of the questionnaire was underlined by only two participants. The 42 items were retained. One item was rephrased on the basis of professionals’ remarks (item A17). The final French version of the questionnaire is provided in (Additional file 1).

### Samples characteristics (Table 2)

The administration of the questionnaire targeted 3,538 professionals. There were 84 “ineligible” professionals. The number of professionals who responded was 2,036. There were 16 incomplete questionnaires. The completion rate for the 2020 questionnaires was 99.04%, and the final participation rate was 58.4%.

**Table 2:** Respondent characteristics for the survey (*n* = 2020)

	French survey	
	n	%
Professional fields		
Paramedical	841	41.6
Administration/logistics/technical	188	9.3
Educational/ Psycho-social	66	3.3
Doctor	59	2.9
Others	41	2.0
Do not wish to answer/missing data	825	40.8
Age bracket		
Under 25 years old	108	5.3
26 to 35 yrs. old	400	19.8
36 to 45 yrs. old	492	24.4
46 to 55 yrs. old	506	25.0
Over 56 yrs. old	146	7.2
Do no wish to answer/missing data	368	18.2
Number of years working in a nursing home		
Less than11 months	170	8.4
1 to 5 years	487	24.1
6 to 10 years	362	17.9
11 years or more	518	25.6
Do not wish to answer/missing data	483	23.9
Weekly working hours in a nursing home		
15 h or fewer	128	6.3
16 to 24 h	132	6.5
25 to 35 h	920	45.5
36 h or more	301	14.9
Do not wish to answer/missing data	539	26.7

Almost half of the respondents (41.6%) belonged to the paramedical field. The age of the professionals was > 35 for 56.6%. Half the respondents had worked in the profession for under 10 years (50.4%). Weekly working hours were 25 to 35 h for the majority (Table 2). 82.1% of the respondents were working in contact with the residents.

### Psychometric validation analysis of the French version of the NHSOPS (Phase 2)

#### First step: test of the original structure

The SEM fit indices were not acceptable (CFI = 0.89; TLI = 0.88; SRMR = 0.06; RMSEA = 0.05 (outputs Additional file 3).

#### Second step: refinement of the original structure

Among the 42 items, 2 were removed because of their missing value rate > 20% (A10 and A17) and one because of ceiling effect (C3).

No pairs of items had Spearman’s correlation coefficients exceeding 0.8 (Additional file 4).

The parallel analysis suggested that seven factors should be retained in the factor analysis (Additional file 5), accounting for 57.1% of the variance after oblique rotation (Table 3). After selecting items with factor loadings exceeding 0.4, the seven factors identified were composed of 22 items. The correlation coefficients between these factors ranged from 0.13 to 0.58 (Additional file 2). The KMO statistic for factor fit was calculated at 0.91, and Bartlett’s test of sphericity was significant (*p* < 0.001).

**Table 3:** Results of the Principal Axis factoring (PAF)

	Factor 1	Factor 2	Factor 3	Factor 4	Factor 5	Factor 6	Factor 7
*Factor loadings*							
A1	0.01	−0.01	0.8	0.05	0.03	0.00	−0.04
A2	0.03	−0.01	0.86	−0.02	−0.02	− 0.01	−0.01
A3	0.09	0.01	0.03	−0.05	−0.09	0.74	0.04
A5	−0.04	0.09	0.57	0.02	0.02	0.04	0.11
A6	0.01	0.05	0.02	0.00	0.73	0.06	−0.05
A8	−0.07	0.00	−0.02	0.06	0.12	0.74	−0.03
A9	−0.1	0.05	0.51	0.05	0.07	0.00	0.13
A14	0.04	−0.02	0.01	0.01	0.72	−0.03	0.08
B1	0.04	0.75	0.06	−0.05	− 0.03	0.03	− 0.03
B2	0.01	0.75	0.05	0.01	0.01	−0.01	−0.02
B3	−0.07	0.67	−0.09	0.04	0.01	0.04	0.05
B5	0.03	0.04	0.02	0.06	0.00	0.04	0.67
B6	−0.03	0.07	0.07	−0.09	0.12	−0.06	0.55
B8	0.26	0.00	0.02	0.17	−0.02	0.04	0.47
B10	0.09	0.62	−0.02	0.05	0.06	−0.06	0.09
C1	0.02	0.04	0.04	0.71	−0.01	0.03	0.08
C2	0.01	−0.01	0.03	0.79	0.01	−0.02	−0.03
D4	0.55	0.11	0.05	0.11	−0.03	0.01	0.02
D5	0.79	0.05	−0.04	0.09	0.01	0.01	0.01
D6	0.88	0.00	0.03	−0.03	−0.02	0.02	0.02
D8	0.67	−0.04	0.04	−0.06	0.15	0.00	0.01
D10	0.40	0.16	−0.1	0.17	0.09	0.03	0.07
*Eigenvalues*							
	7.20	1.66	1.09	0.94	0.78	0.55	0.36
*Percentage of variance accounted for*							
%	32.7	7.5	4.9	4.3	3.5	2.5	1.6
Cumulative %	32.7	40.3	45.2	49.5	53	55.5	57.1

The NHSOPS-F was thus composed of 22 items and seven factors, which correspond, in part, to dimensions in the NHSOPS: Teamwork (A1, A2, A5, A9), Staffing (A3, A8), Compliance with procedures (A6, A14), Handoffs (B1, B2, B3, B10), Feedback and communication about incidents (B5, B6, B8), Supervisor expectations and actions promoting resident safety (C1, C2), and Overall perceptions of resident safety and organizational learning (D4, D5, D6, D8, D10) (Table 3). The description of answers according to response choices are presented in (Additional file 6).

The SEM on the remaining third of the data confirmed the existence of the seven latent factors. All the indices indicated a good fit to the data (CFI = 0.960; TLI = 0.951; SRMR = 0.044; RMSEA = 0.046) and the structural coefficients were highly significant (*P* < 0.001) (Additional files 7 and 8).

Cronbach’s α-coefficients were > 0.70 (Table 4). Twenty-one professionals answered the questionnaire twice. The ICC per item ranged from 0.19 to 0.88 (Table 5).

**Table 4:** Cronbach’s α-coefficients for the seven dimensions retained

Dimensions (*n* = 7) (corresponding factors)	Cronbach’s α-coefficients	
Overall perceptions of resident safety and Organizational learning (factor 1)	0.865	
Handoffs (transfer of information) (factor 2)	0.824	
Teamwork (factor 3)	0.832	
Supervisor expectations and actions promoting resident safety (factor 4)	0.745	
Compliance with procedures (factor 5)	0.720	
Staffing (factor 6)	0.738	
Feedback and communication about incidents (factor 7)	0.727

**Table 5:** Intra-class correlation (ICC) per item

Dimensions (*n* = 7)	Items	Intra-class correlation
Overall perceptions of resident safety and Organizational learning		
	D4	0.61
	D5	0.76
	D6	0.82
	D8	0.54
	D10	0.37
Handoffs (transfer of information)		
	B1	0.82
	B2	0.57
	B3	0.88
	B10	0.60
Teamwork		
	A1	0.51
	A2	0.70
	A5	0.43
	A9	0.52
Supervisor expectations and actions promoting resident safety		
	C1	0.58
	C2	0.45
Compliance with procedures		
	A14	0.64
	A6	0.65
Staffing		
	A3	0.81
	A8	0.86
Feedback and communication about incidents		
	B5	0.76
	B6	0.59
	B8	0.19

## Discussion

The psychometric properties of the NHSOPS were reported by the American authors to be satisfactory [21]. These authors suggested that the scale could be used internationally, and they produced a document to guide transcultural adaptation procedures and questionnaire administration [20]. These various recommendations were complied with in the present study.

The first step consisting in the translation and transcultural adaptation culminated in the elaboration of the French version of the NHSOPS, which maintains the original 42 items. The adaptation of the questionnaire in other countries had produced versions comprising 42 [25, 27] or 43 items [24] (one extra item in the “Handoffs” domain).

The second stage of psychometric validation was then conducted, concluding to a seven-factor structure. The factor (dimension) “Teamwork” returns to the 4 original items in this domain and explores support, mutual respect and collaboration among colleagues. The dimension “Staffing” explores staffing numbers in relation to workload (2 items maintained from the 4 original items). The dimension “Compliance with procedures” explores general compliance with the mandatory procedures in the facility whatever the functioning difficulties encountered by professionals (2 items out of the original 3). The dimension “Handoffs” maintains the 4 initial items, and assesses communication for resident care within the EHPAD and with outside structures. The dimension “Feedback and communication about incidents” explores the way in which potential or actual incidents are dealt with, and the sharing of solutions (3 items out of the initial 4). The dimension “Supervisor expectations and actions promoting resident safety” explores the valorisation of professional compliance with procedures and receptivity by supervisors towards ideas for improvement (2 of the 3 original items). The dimension “Overall perceptions of resident safety and Organizational learning” comprises 5 of the original 7 items derived from 2 initial dimensions. This theme explores the ability of the facility to take account of its mistakes and make changes, and the overall quality of care provided for residents.

In our model, the number of items included was 22. These items initially belonged to 8 dimensions in the original American questionnaire. The factor analysis on the seven factors retained accounted for 57.1% of the variance.

In studies in other countries, the exploratory factor analyses conducted led to a reduction in the number of dimensions from 12 to 9 for Switzerland [25] and 10 for Norway [24]. The numbers of items retained were respectively 42 and 32. Explained variance was 58.1% for the Swiss study, while this result was not presented in the Norwegian study. For China, Lin et al. presented a final model comprising 4 dimensions and 29 items [27]. The explained variance reached 52.8%.

These different studies have produced different models for the NHSOPS. The advantage of our model resides in the fact that i) the number of factors is sufficient to claim precision in the structure of the model, ii) our model was constructed from a fairly large database (*n* = 2020 questionnaires and 61 EHPAD facilities). This database was considerably larger than that found in studies in other countries (*n* = 466 questionnaires from 12 establishments in Norway [24], *n* = 367 questionnaires and 9 establishments in Switzerland [25] and *n* = 306 questionnaires from 30 establishments in China [27]). Our methodology was exactly the same as that used by Lin et al., but their smaller sample size did not enable them to achieve a model as refined as ours. It can also be noted that these studies in different countries targeted certain professional categories (managers [27], only professionals directly involved in care [25]), which, as acknowledged by the authors, was a limitation of their data. It can be recalled that the AHRQ recommends soliciting all professionals in nursing homes to respond to the survey [21]. The sample of professionals in our study was for its part representative of the population working in EHPAD facilities in France [37].

Finally, it can be noted that the participation rate among EHPAD professionals in our study was 58.4%. The rates in the other countries were 66% [25], 69% [24], and 100% [27]. The participation rates are not comparable because of the differences in the professional populations targeted. Because of the mode of administration of the questionnaires, reasons for non-participation in our study are not known.

Generally speaking, there is no consensus across countries on the definition of the safety culture (SC) and the dimensions that make it up (in terms of number, content and denomination) [38]. In France, the HAS (French health authority) retains the definition of the European Society for the Quality of Health Care: “a coherent and integrated system of individual and organisational behaviours, based on shared beliefs and values, continuously striving to reduce damage caused to patients potentially linked to patient care procedures” [11]. In France the HAS also recommends the exploration of 10 dimensions of the SC in care provision. They are as follows: teamwork within the department, teamwork between departments, human resources, non punitive response to mistakes, frequency of notification of adverse events, freedom of expression, management support for care safety, expectations and actions by management for care safety, learning and continuous improvement, global perceptions of safety.

If we compare these 10 dimensions generally used in France with the 7 dimensions in our model, six explicitly coincide – teamwork within the department (teamwork), teamwork between departments (handoffs), human resources (staffing), expectation and actions by management for care safety, learning and continuous improvement, global perceptions of safety [11]. One dimension is not explored in our model, since it was not initially included in the AHRQ version of the NHSOPS – the “frequency of notification of adverse events”. Finally, three dimensions are not found explicitly, but their general themes are found in items C1, C2, D4, D5 and D10 (item labels provided in Additional file 1). Likewise, “freedom of expression” and “non-punitive response to mistakes” are contained in items B5, B6 and B8.

One limitation of our study is found in our test-retest results. Certain items have a very low ICC. Polit [39] in a recent article highlighted the importance of the sampling procedure in this type of test, and in particular the need for heterogeneity among respondents (there was little heterogeneity in our sample), and the need for a sample of at least 50 (only 21 in our study). Polit also suggests detailed comparison of responses to each item between the two administrations, coupled with in-depth interviews of certain respondents to explore the coherence of responses. The test-retest will be reiterated before the finalisation of the scale (as is indeed suggested by Polit [39]), within a study conducted on an EHPAD sample extended to the national territory as a whole. The checklists for the design of reliability tests will be taken into account [39]. This national study should confirm or improve the structure of our model. The 22 items in the model will be maintained in the NHSOPS-F questionnaire. Professionals will also be invited to respond to extra items (relating to the frequency of notification of adverse events) and to original items maintained for pedagogical purposes at the time of feedback on results to the professionals. It should indeed be remembered that the NHSOPS questionnaire gives professionals the opportunity to exchange views on SC in nursing homes in the setting of the deployment of risk management in healthcare [20].

## Conclusion

The NHSOPS questionnaire, presented here in its French language version, is the first questionnaire on Patient SC that has been applied to the medico-social sector in France. The analysis of its psychometric properties has enabled the validation of seven dimensions. One limitation of our study is found in our test-retest results. This instrument was developed from a large database. An upcoming national survey in the second semester of 2019 should contribute to confirming or improving the structure of our model. In the meantime, the NHSOPS questionnaire gives professionals the opportunity to exchange viewpoints on SC in nursing homes in the context of the deployment of risk management in healthcare. This study is part of a research project [28], the results of which will enable programmes of support and awareness-raising on these SC issues in nursing homes to be designed. These assessment tools come within the scope of the continuing promotion of healthcare safety endorsed by healthcare policies and supported by recent institutional and regulatory evolutions in the area of healthcare safety.

## Additional files

Additional file 1: a. Nursing Home Survey on Patient Safety. b. French version of the NHSOPS questionnaire. (ZIP 334 kb)

Additional file 2: Inter-correlations across the 7 final dimensions retained. (PDF 91 kb)

Additional file 3: Original SEM outputs. (PDF 103 kb)

Additional file 4: Inter-correlations across the 42 items of the questionnaire . (PDF 186 kb)

Additional file 5: Parallel analysis scree plots. (PNG 12 kb)

Additional file 6: Description of item responses per answer choice (percentages) . (PDF 171 kb)

Additional file 7: Structural Equation Model outputs. (PDF 237 kb)

Additional file 8: Structural Equation Model diagram. (PNG 28 kb)

## Data Availability

Please contact the corresponding author should you require any of the results from this study.

## Abbreviations

AHRQ: Agency for Healthcare Research and Quality
CFI: Comparative Fit Index
DGOS: Direction Général de l’Offre des Soins
EHPAD: Etablissements d’Hébergement pour Personnes Agées Dépendantes
HSOSC: Hospital Survey on Patient Safety Culture
ICC: Intra-class Correlation Coefficients
IRB: Institutional Review Board
NHSOPS: Nursing Home Survey on Patient Safety Culture
RMSEA: Steiger’s Root Mean Square Error of Approximation
SC: Safety Culture
SEM: Structural Equation Model
SRMR: Standardized Root Mean Square Residual
TLI: Tucker Lewis Index
