# Supplementary material for: Transcultural adaptation and psychometric study of the French version of the nursing home survey on patient safety culture questionnaire
Source: BMC Health Serv Res. 2019 Jul 15;19:490. doi: 10.1186/s12913-019-4333-5 (PMC6631961; doi:10.1186/s12913-019-4333-5)
Supplement: Supplementary file 1 — a. Nursing Home Survey on Patient Safety. b. French version of the NHSOPS questionnaire. (ZIP 334 kb) [file 12913_2019_4333_MOESM1_ESM.zip › Additional file 1a_nhsurveyR5.pdf]

In this survey, “resident safety” means preventing resident injuries, incidents, and harm to residents in the nursing home.

This survey asks for your opinions about resident safety issues in your nursing home. It will take about 15 minutes to complete.

To mark your answer, just put an X or a √ in the box: x or √.

If a question does not apply to your job or you do not know the answer, please mark the box in the last column. If you do not wish to answer a question, you may leave your answer blank.

SECTION A: Working in This Nursing Home

How much do you agree or disagree with the following statements?	Strongly Disagree ▼	Disagree ▼	Neither Agree nor Disagree ▼	Agree ▼	Strongly Agree ▼	Does Not Apply or Don’t Know ▼
1. Staff in this nursing home treat each other with respect	☐1	☐2	☐3	☐4	☐5	☐9
2. Staff support one another in this nursing home	☐1	☐2	☐3	☐4	☐5	☐9
3. We have enough staff to handle the workload	☐1	☐2	☐3	☐4	☐5	☐9
4. Staff follow standard procedures to care for residents	☐1	☐2	☐3	☐4	☐5	☐9
5. Staff feel like they are part of a team	☐1	☐2	☐3	☐4	☐5	☐9
6. Staff use shortcuts to get their work done faster	☐1	☐2	☐3	☐4	☐5	☐9
7. Staff get the training they need in this nursing home	☐1	☐2	☐3	☐4	☐5	☐9
8. Staff have to hurry because they have too much work to do	☐1	☐2	☐3	☐4	☐5	☐9
9. When someone gets really busy in this nursing home, other staff help out	☐1	☐2	☐3	☐4	☐5	☐9
10. Staff are blamed when a resident is harmed	☐1	☐2	☐3	☐4	☐5	☐9

SECTION A: Working in This Nursing Home (continued)

	Strongly Disagree ▼	Disagree ▼	Neither Agree nor Disagree ▼	Agree ▼	Strongly Agree ▼	Does Not Apply or Don't Know ▼
11. Staff have enough training on how to handle difficult residents	☐1	☐2	☐3	☐4	☐5	☐9
12. Staff are afraid to report their mistakes	☐1	☐2	☐3	☐4	☐5	☐9
13. Staff understand the training they get in this nursing home	☐1	☐2	☐3	☐4	☐5	☐9
14. To make work easier, staff often ignore procedures	☐1	☐2	☐3	☐4	☐5	☐9
15. Staff are treated fairly when they make mistakes	☐1	☐2	☐3	☐4	☐5	☐9
16. Residents' needs are met during shift changes	☐1	☐2	☐3	☐4	☐5	☐9
17. It is hard to keep residents safe here because so many staff quit their jobs	☐1	☐2	☐3	☐4	☐5	☐9
18. Staff feel safe reporting their mistakes	☐1	☐2	☐3	☐4	☐5	☐9

SECTION B: Communications

How often do the following things happen in your nursing home?	Never ▼	Rarely ▼	Some-times ▼	Most of the time ▼	Always ▼	Does Not Apply or Don't Know ▼
1. Staff are told what they need to know before taking care of a resident for the first time	☐1	☐2	☐3	☐4	☐5	☐9
2. Staff are told right away when there is a change in a resident's care plan	☐1	☐2	☐3	☐4	☐5	☐9
3. We have all the information we need when residents are transferred from the hospital	☐1	☐2	☐3	☐4	☐5	☐9
4. When staff report something that could harm a resident, someone takes care of it	☐1	☐2	☐3	☐4	☐5	☐9
5. In this nursing home, we talk about ways to keep incidents from happening again	☐1	☐2	☐3	☐4	☐5	☐9

SECTION B: Communications (continued)

	Never	Rarely	Some-times	Most of the time	Always	Does Not Apply or Don't Know
6. Staff tell someone if they see something that might harm a resident	☐1	☐2	☐3	☐4	☐5	☐9
7. Staff ideas and suggestions are valued in this nursing home	☐1	☐2	☐3	☐4	☐5	☐9
8. In this nursing home, we discuss ways to keep residents safe from harm	☐1	☐2	☐3	☐4	☐5	☐9
9. Staff opinions are ignored in this nursing home	☐1	☐2	☐3	☐4	☐5	☐9
10. Staff are given all the information they need to care for residents	☐1	☐2	☐3	☐4	☐5	☐9
11. It is easy for staff to speak up about problems in this nursing home	☐1	☐2	☐3	☐4	☐5	☐9

SECTION C: Your Supervisor

How much do you agree or disagree with the following statements?	Strongly Disagree	Disagree	Neither Agree nor Disagree	Agree	Strongly Agree	Does Not Apply or Don't Know
1. My supervisor listens to staff ideas and suggestions about resident safety	☐1	☐2	☐3	☐4	☐5	☐9
2. My supervisor says a good word to staff who follow the right procedures	☐1	☐2	☐3	☐4	☐5	☐9
3. My supervisor pays attention to resident safety problems in this nursing home	☐1	☐2	☐3	☐4	☐5	☐9

SECTION D: Your Nursing Home

How much do you agree or disagree with the following statements?	Strongly Disagree	Disagree	Neither Agree nor Disagree	Agree	Strongly Agree	Does Not Apply or Don't Know
1. Residents are well cared for in this nursing home	☐1	☐2	☐3	☐4	☐5	☐9
2. Management asks staff how the nursing home can improve resident safety	☐1	☐2	☐3	☐4	☐5	☐9
3. This nursing home lets the same mistakes happen again and again	☐1	☐2	☐3	☐4	☐5	☐9

SECTION D: Your Nursing Home (continued)

	Strongly Disagree ▼	Disagree ▼	Neither Agree nor Disagree ▼	Agree ▼	Strongly Agree ▼	Does Not Apply or Don't Know ▼
4. It is easy to make changes to improve resident safety in this nursing home	☐1	☐2	☐3	☐4	☐5	☐9
5. This nursing home is always doing things to improve resident safety	☐1	☐2	☐3	☐4	☐5	☐9
6. This nursing home does a good job keeping residents safe	☐1	☐2	☐3	☐4	☐5	☐9
7. Management listens to staff ideas and suggestions to improve resident safety	☐1	☐2	☐3	☐4	☐5	☐9
8. This nursing home is a safe place for residents	☐1	☐2	☐3	☐4	☐5	☐9
9. Management often walks around the nursing home to check on resident care........	☐1	☐2	☐3	☐4	☐5	☐9
10. When this nursing home makes changes to improve resident safety, it checks to see if the changes worked	☐1	☐2	☐3	☐4	☐5	☐9

SECTION E: Overall Ratings

1. I would tell friends that this is a safe nursing home for their family.
   - ☐ a. Yes
   - ☐ b. Maybe
   - ☐ c. No

2. Please give this nursing home an overall rating on resident safety.

Poor ▼	Fair ▼	Good ▼	Very good ▼	Excellent ▼
☐1	☐2	☐3	☐4	☐5

SECTION F: Background Information

1. What is your job in this nursing home? Check ONE box that best applies to your job. If more than one category applies, check the highest level job.

- ☐ a. Administrator/Manager
  - Executive Director/Administrator
  - Medical Director
  - Director of Nursing/Nursing Supervisor
  - Department Head
  - Unit Manager/Charge Nurse
  - Assistant Director/Assistant Manager
  - Minimum Data Set (MDS) Coordinator/ Resident Nurse Assessment Coordinator (RNAC)
- ☐ b. Physician (MD, DO)
- ☐ c. Other Provider
  - Nurse Practitioner
  - Clinical Nurse Specialist
  - Physician Assistant
- ☐ d. Licensed Nurse
  - Registered Nurse (RN)
  - Licensed Practical Nurse (LPN)
  - Wound Care Nurse
- ☐ e. Nursing Assistant/Aide
  - Certified Nursing Assistant (CNA)
  - Geriatric Nursing Assistant (GNA)
  - Nursing Aide/Nursing Assistant
- ☐ f. Direct Care Staff
  - Activities Staff Member
  - Dietitian/Nutritionist
  - Medication Technician
  - Pastoral Care/Chaplain
  - Pharmacist
  - Physical/Occupational/Speech/ Respiratory Therapist
  - Podiatrist
  - Social Worker
- ☐ g. Administrative Support Staff
  - Administrative Assistant
  - Admissions
  - Billing/Insurance
  - Secretary
  - Human Resources
  - Medical Records
- ☐ h. Support Staff
  - Drivers
  - Food Service/Dietary
  - Housekeeping
  - Laundry Service
  - Maintenance
  - Security
- ☐ i. Other (Please write the title of your job):

  ____

2. How long have you worked in this nursing home?

- ☐ a. Less than 2 months
- ☐ b. 2 to 11 months
- ☐ c. 1 to 2 years
- ☐ d. 3 to 5 years
- ☐ e. 6 to 10 years
- ☐ f. 11 years or more

3. How many hours per week do you usually work <u>in this nursing home</u>?

- ☐ a. 15 or fewer hours per week
- ☐ b. 16 to 24 hours per week
- ☐ c. 25 to 40 hours per week
- ☐ d. More than 40 hours per week

SECTION F: Background Information (continued)

4. When do you work most often? Check ONE answer.
   - ☐ a. Days
   - ☐ b. Evenings
   - ☐ c. Nights

5. Are you paid by a staffing agency when you work for this nursing home?
   - ☐ a. Yes
   - ☐ b. No

6. In your job in this nursing home, do you work directly with residents most of the time? Check ONE answer.
   - ☐ a. YES, I work directly with residents most of the time.
   - ☐ b. NO, I do NOT work directly with residents most of the time.

7. In this nursing home, where do you spend most of your time working? Check ONE answer.
   - ☐ a. Many different areas or units in this nursing home / No specific area or unit
   - ☐ b. Alzheimer's / Dementia unit
   - ☐ c. Rehab unit
   - ☐ d. Skilled nursing unit
   - ☐ e. Other area or unit (Please specify): ______________________________

SECTION G: Your Comments

Please feel free to write any comments about resident care and safety in this nursing home.

THANK YOU FOR COMPLETING THIS SURVEY.
