# Supplementary material for: Transcultural adaptation and psychometric study of the French version of the nursing home survey on patient safety culture questionnaire
Source: BMC Health Serv Res. 2019 Jul 15;19:490. doi: 10.1186/s12913-019-4333-5 (PMC6631961; doi:10.1186/s12913-019-4333-5)
Supplement: Supplementary file 1 — a. Nursing Home Survey on Patient Safety. b. French version of the NHSOPS questionnaire. (ZIP 334 kb) [file 12913_2019_4333_MOESM1_ESM.zip › Additional file 1b_frenchsurveyR5.pdf]

Additional file 1b: French version of the NHSOPS

A. Le travail dans votre établissement

Dans quelle mesure êtes-vous en accord ou en désaccord avec les affirmations suivantes?		Pas du tout d'accord ▼	Pas d'accord ▼	Moyennement d'accord ▼	D'accord ▼	Tout à fait d'accord ▼	Ne sais pas ou non applicable ▼
A1	Les membres du personnel de cet établissement se respectent les uns les autres.	☐1	☐2	☐3	☐4	☐5	☐9
A2	Les membres du personnel de cet établissement se soutiennent entre eux.	☐1	☐2	☐3	☐4	☐5	☐9
A3	Nous avons suffisamment de personnel pour faire face à la charge de travail.	☐1	☐2	☐3	☐4	☐5	☐9
A4	Le personnel suit les procédures de prise en charge des résidents.	☐1	☐2	☐3	☐4	☐5	☐9
A5	Les membres du personnel du service ont le sentiment de faire partie d'une équipe.	☐1	☐2	☐3	☐4	☐5	☐9
A6	Pour accomplir ses tâches plus rapidement, le personnel ne respecte pas l'ensemble des procédures.	☐1	☐2	☐3	☐4	☐5	☐9
A7	Le personnel de cet établissement reçoit les formations dont il a besoin.	☐1	☐2	☐3	☐4	☐5	☐9
A8	Les membres du personnel doivent se dépêcher car ils ont trop de travail.	☐1	☐2	☐3	☐4	☐5	☐9
A9	Lorsque quelqu'un a trop de travail, les autres membres de l'équipe lui viennent en aide.	☐1	☐2	☐3	☐4	☐5	☐9
A10	En cas de dommage subi par un résident, le personnel est tenu pour responsable.	☐1	☐2	☐3	☐4	☐5	☐9
A11	Le personnel est suffisamment formé pour prendre en charge les résidents difficiles.	☐1	☐2	☐3	☐4	☐5	☐9
A12	Le personnel a peur de signaler ses erreurs.	☐1	☐2	☐3	☐4	☐5	☐9
A13	Le personnel comprend les formations suivies dans l'établissement.	☐1	☐2	☐3	☐4	☐5	☐9

A. Le travail dans votre établissement (Suite)

		Pas du tout d'accord	Pas d'accord	Moyennement d'accord	D'accord	Tout à fait d'accord	Ne sais pas ou non applicable
A14.	Pour faciliter son travail, le personnel contourne souvent les procédures.	☐1	☐2	☐3	☐4	☐5	☐9
A15.	Le personnel est traité de façon appropriée quand il commet une erreur.	☐1	☐2	☐3	☐4	☐5	☐9
A16.	Les besoins des résidents sont satisfaits durant les changements d'équipe.	☐1	☐2	☐3	☐4	☐5	☐9
A17.	Il est difficile d'assurer la sécurité des résidents du fait d'un grand nombre de départs volontaires (ex. démissions…).	☐1	☐2	☐3	☐4	☐5	☐9
A18.	Le personnel a le sentiment de pouvoir signaler ses erreurs en toute sécurité.	☐1	☐2	☐3	☐4	☐5	☐9

B. Communications

<u>A quelle fréquence</u> les situations suivantes surviennent-elles dans votre établissement?

		Jamais	Rarement	Quelque-fois	La plupart du temps	Toujours	Ne sais pas ou non applicable
B1.	Avant de s'occuper d'un résident pour la première fois, le personnel reçoit toutes les informations dont il a besoin.	☐1	☐2	☐3	☐4	☐5	☐9
B2.	Le personnel est informé immédiatement lorsqu'un changement survient dans le plan de prise en charge d'un résident.	☐1	☐2	☐3	☐4	☐5	☐9
B3.	Le personnel dispose de toutes les informations nécessaires lorsqu'un résident revient de l'hôpital.	☐1	☐2	☐3	☐4	☐5	☐9
B4.	Quand le personnel signale quelque chose qui pourrait nuire à un résident, une personne s'en occupe.	☐1	☐2	☐3	☐4	☐5	☐9
B5.	Dans cet établissement, nous réfléchissons ensemble aux moyens d'éviter qu'un incident ne se reproduise.	☐1	☐2	☐3	☐4	☐5	☐9
B6.	S'ils voient quelque chose qui pourrait nuire à un résident, les membres du personnel en parlent à une personne.	☐1	☐2	☐3	☐4	☐5	☐9
B7.	Cet établissement attache de l'importance au partage des idées et aux suggestions du personnel.	☐1	☐2	☐3	☐4	☐5	☐9
B8.	Dans cet établissement, nous discutons de la façon d'assurer la protection des résidents.	☐1	☐2	☐3	☐4	☐5	☐9
B9.	Cet établissement ne tient pas compte de l'opinion du personnel.	☐1	☐2	☐3	☐4	☐5	☐9

B10.	Le personnel reçoit toute l'information nécessaire à la prise en charge des résidents.	☐1	☐2	☐3	☐4	☐5	☐9
B11.	Il est facile pour le personnel de parler ouvertement des problèmes dans cet établissement.	☐1	☐2	☐3	☐4	☐5	☐9

C. Votre responsable

	Dans quelle mesure êtes-vous en accord ou en désaccord avec les affirmations suivantes?	Jamais ▼	Rarement ▼	Quelque fois ▼	La plupart du temps ▼	Toujours ▼	Ne sais pas ou non applicable ▼
C1.	Mon responsable est à l'écoute des idées et suggestions du personnel concernant la sécurité des résidents.	☐1	☐2	☐3	☐4	☐5	☐9
C2.	Mon responsable encourage le personnel qui respecte les procédures.	☐1	☐2	☐3	☐4	☐5	☐9
C3.	Mon responsable est attentif aux problèmes de sécurité des résidents dans l'établissement.	☐1	☐2	☐3	☐4	☐5	☐9

D. Votre établissement

	Dans quelle mesure êtes-vous en accord ou en désaccord avec les affirmations suivantes?	Jamais ▼	Rarement ▼	Quelque fois ▼	La plupart du temps ▼	Toujours ▼	Ne sais pas ou non applicable ▼
D1.	On prend bien soin des résidents dans cet établissement.	☐1	☐2	☐3	☐4	☐5	☐9
D2.	La direction demande au personnel comment améliorer la sécurité des résidents de l'établissement.	☐1	☐2	☐3	☐4	☐5	☐9
D3.	Cet établissement laisse les mêmes erreurs se reproduire encore et encore.	☐1	☐2	☐3	☐4	☐5	☐9
D4.	Dans cet établissement, il est facile d'apporter des changements visant à améliorer la sécurité des résidents.	☐1	☐2	☐3	☐4	☐5	☐9
D5.	Cet établissement agit constamment en faveur de l'amélioration de la sécurité des résidents.	☐1	☐2	☐3	☐4	☐5	☐9
D6.	Cet établissement réalise un bon travail pour assurer la sécurité des résidents	☐1	☐2	☐3	☐4	☐5	☐9
D7.	La direction est à l'écoute des idées et suggestions du personnel pour améliorer la sécurité des résidents.	☐1	☐2	☐3	☐4	☐5	☐9
D8.	Cet établissement est un endroit sûr pour les résidents.	☐1	☐2	☐3	☐4	☐5	☐9

D9.	La direction fait souvent le tour de l'établissement pour apprécier les soins donnés aux résidents.	☐1	☐2	☐3	☐4	☐5	☐9
D10.	Lorsque des actions d'amélioration de la sécurité des résidents sont mises en place, l'établissement vérifie qu'elles ont bien fonctionné.	☐1	☐2	☐3	☐4	☐5	☐9

E. Evaluation globale

E1. Je pourrais dire à mes amis que c'est un établissement sûr pour les membres de leur famille.

☐ Oui ☐ Peut-être ☐ Non

E2. Globalement, comment jugez-vous la sécurité des résidents dans votre établissement ?

☐ Faible ☐ Moyenne ☐ Bonne ☐ Très bonne ☐ Excellente
