# Supplementary material for: Transcultural adaptation and psychometric study of the French version of the nursing home survey on patient safety culture questionnaire
Source: BMC Health Serv Res. 2019 Jul 15;19:490. doi: 10.1186/s12913-019-4333-5 (PMC6631961; doi:10.1186/s12913-019-4333-5)
Supplement: Supplementary file 2 — Inter-correlations across the 7 final dimensions retained. (PDF 91 kb) [file 12913_2019_4333_MOESM2_ESM.pdf]

Additional file 2

Table: Correlation coefficients across the seven principal factors of the exploratory factor analysis

	1	2	3	4	5	6	7
Factor 1	1.00						
Factor 2	0.54	1.00					
Factor 3	0.26	0.35	1.00				
Factor 4	0.56	0.48	0.35	1.00			
Factor 5	0.32	0.36	0.32	0.21	1.00		
Factor 6	0.34	0.27	0.15	0.21	0.26	1.00	
Factor 7	0.59	0.58	0.35	0.56	0.33	0.13	1.00
