# Supplementary material for: Transcultural adaptation and psychometric study of the French version of the nursing home survey on patient safety culture questionnaire
Source: BMC Health Serv Res. 2019 Jul 15;19:490. doi: 10.1186/s12913-019-4333-5 (PMC6631961; doi:10.1186/s12913-019-4333-5)
Supplement: Supplementary file 3 — Original SEM outputs. (PDF 103 kb) [file 12913_2019_4333_MOESM3_ESM.pdf]

Additional file 3

Table : Indicators to assess the global fit of the original structural equation model (R output)

npar	fmin	chisq	df	pvalue	baseline.chisq
150.000	1.574	2313.325	753.000	0.000	15527.471
baseline.df	baseline.pvalue	cfi	tli	nnfi	rfi
861.000	0.000	0.894	0.878	0.878	0.830
nfi	pnfi	ifi	rni	logl	unrestricted.logl
0.851	0.744	0.894	0.894	-33132.802	-31974.566
aic	bic	ntotal	bic2	rmsea	rmsea.ci.lower
66565.604	67255.789	736.000	66779.488	0.053	0.051
rmsea.ci.upper	rmsea.pvalue	rmr	rmr_nomean	srmr	srmr_bentler
0.056	0.019	0.045	0.045	0.057	0.057
srmr_bentler_nomean	crmr	crmr_nomean	srmr_mplus	srmr_mplus_nomean	cn_05
0.057	0.058	0.058	0.057	0.057	260.882
cn_01	gfi	agfi	pgfi	mfi	ecvi
269.861	0.864	0.836	0.720	0.346	3.556
