# Supplementary material for: Transcultural adaptation and psychometric study of the French version of the nursing home survey on patient safety culture questionnaire
Source: BMC Health Serv Res. 2019 Jul 15;19:490. doi: 10.1186/s12913-019-4333-5 (PMC6631961; doi:10.1186/s12913-019-4333-5)
Supplement: Supplementary file 4 — Inter-correlations across the 42 items of the questionnaire . (PDF 186 kb) [file 12913_2019_4333_MOESM4_ESM.pdf]

Additional file 4

Table: Spearman's correlation coefficients between the 42 items

| | A1 | A2 | A3 | A4 | A5 | A6 | A7 | A8 | A9 | A10 | A11 | A12 | A13 | A14 | A15 | A16 | A17 | A18 | B1 | B2 | B3 | B4 | B5 | B6 | B7 | B8 | B9 | B10 | B11 | C1 | C2 | C3 | D1 | D2 | D3 | D4 | D5 | D6 | D7 | D8 | D9 | D10 |
|---|---|---|---|---|---|---|---|---|---|---|---|---|---|---|---|---|---|---|---|---|---|---|---|---|---|---|---|---|---|---|---|---|---|---|---|---|---|---|---|---|---|---|
| **A1** | 1,00 | 0,68 | 0,08 | 0,33 | 0,53 | 0,20 | 0,07 | 0,03 | 0,43 | 0,06 | 0,10 | 0,19 | 0,21 | 0,22 | 0,20 | 0,24 | 0,15 | 0,24 | 0,22 | 0,21 | 0,12 | 0,23 | 0,21 | 0,22 | 0,24 | 0,21 | 0,19 | 0,20 | 0,27 | 0,21 | 0,22 | 0,19 | 0,25 | 0,11 | 0,29 | 0,19 | 0,23 | 0,25 | 0,20 | 0,19 | 0,16 | 0,16 |
| **A2** | 0,68 | 1,00 | 0,07 | 0,29 | 0,49 | 0,20 | 0,11 | 0,05 | 0,47 | 0,05 | 0,10 | 0,18 | 0,22 | 0,18 | 0,23 | 0,22 | 0,16 | 0,25 | 0,22 | 0,24 | 0,11 | 0,22 | 0,22 | 0,24 | 0,20 | 0,19 | 0,16 | 0,21 | 0,30 | 0,19 | 0,23 | 0,18 | 0,24 | 0,10 | 0,23 | 0,19 | 0,20 | 0,23 | 0,16 | 0,19 | 0,14 | 0,13 |
| **A3** | 0,08 | 0,07 | 1,00 | 0,21 | 0,11 | 0,14 | 0,27 | 0,62 | 0,08 | 0,04 | 0,33 | 0,04 | 0,11 | 0,14 | 0,20 | 0,25 | 0,30 | 0,20 | 0,26 | 0,15 | 0,15 | 0,23 | 0,17 | 0,04 | 0,29 | 0,20 | 0,22 | 0,19 | 0,22 | 0,20 | 0,13 | 0,18 | 0,24 | 0,29 | 0,24 | 0,24 | 0,28 | 0,30 | 0,33 | 0,27 | 0,33 | 0,25 |
| **A4** | 0,33 | 0,29 | 0,21 | 1,00 | 0,40 | 0,40 | 0,23 | 0,14 | 0,29 | 0,07 | 0,23 | 0,23 | 0,24 | 0,46 | 0,14 | 0,32 | 0,18 | 0,21 | 0,23 | 0,27 | 0,18 | 0,24 | 0,35 | 0,29 | 0,25 | 0,31 | 0,10 | 0,26 | 0,24 | 0,20 | 0,18 | 0,22 | 0,37 | 0,22 | 0,32 | 0,22 | 0,31 | 0,36 | 0,24 | 0,36 | 0,24 | 0,32 |
| **A5** | 0,53 | 0,49 | 0,11 | 0,40 | 1,00 | 0,27 | 0,20 | 0,08 | 0,42 | 0,10 | 0,18 | 0,26 | 0,34 | 0,28 | 0,26 | 0,27 | 0,23 | 0,29 | 0,27 | 0,29 | 0,19 | 0,24 | 0,26 | 0,29 | 0,29 | 0,27 | 0,24 | 0,24 | 0,29 | 0,22 | 0,24 | 0,20 | 0,23 | 0,15 | 0,29 | 0,22 | 0,23 | 0,25 | 0,24 | 0,22 | 0,19 | 0,22 |
| **A6** | 0,20 | 0,20 | 0,14 | 0,40 | 0,27 | 1,00 | 0,09 | 0,27 | 0,21 | 0,17 | 0,16 | 0,33 | 0,16 | 0,58 | 0,13 | 0,28 | 0,21 | 0,16 | 0,21 | 0,24 | 0,14 | 0,16 | 0,19 | 0,21 | 0,21 | 0,24 | 0,21 | 0,26 | 0,17 | 0,18 | 0,18 | 0,20 | 0,35 | 0,18 | 0,32 | 0,20 | 0,25 | 0,26 | 0,20 | 0,27 | 0,22 | 0,27 |
| **A7** | 0,07 | 0,11 | 0,27 | 0,23 | 0,20 | 0,09 | 1,00 | 0,19 | 0,08 | 0,03 | 0,42 | 0,07 | 0,34 | 0,18 | 0,29 | 0,18 | 0,19 | 0,26 | 0,27 | 0,22 | 0,16 | 0,28 | 0,29 | 0,16 | 0,37 | 0,33 | 0,24 | 0,27 | 0,26 | 0,30 | 0,28 | 0,31 | 0,20 | 0,38 | 0,31 | 0,29 | 0,39 | 0,34 | 0,40 | 0,28 | 0,31 | 0,37 |
| **A8** | 0,03 | 0,05 | 0,62 | 0,14 | 0,08 | 0,27 | 0,19 | 1,00 | 0,06 | 0,13 | 0,27 | 0,11 | 0,05 | 0,19 | 0,21 | 0,24 | 0,29 | 0,17 | 0,23 | 0,21 | 0,21 | 0,22 | 0,16 | 0,03 | 0,25 | 0,19 | 0,20 | 0,23 | 0,21 | 0,20 | 0,17 | 0,21 | 0,26 | 0,25 | 0,26 | 0,21 | 0,24 | 0,24 | 0,28 | 0,24 | 0,28 | 0,23 |
| **A9** | 0,43 | 0,47 | 0,08 | 0,29 | 0,42 | 0,21 | 0,08 | 0,06 | 1,00 | 0,03 | 0,13 | 0,20 | 0,20 | 0,25 | 0,20 | 0,20 | 0,14 | 0,22 | 0,24 | 0,26 | 0,20 | 0,22 | 0,24 | 0,25 | 0,26 | 0,22 | 0,11 | 0,24 | 0,21 | 0,23 | 0,20 | 0,17 | 0,22 | 0,11 | 0,21 | 0,19 | 0,15 | 0,18 | 0,18 | 0,17 | 0,14 | 0,16 |
| **A10** | 0,06 | 0,05 | 0,04 | 0,07 | 0,10 | 0,17 | 0,03 | 0,13 | 0,03 | 1,00 | 0,03 | 0,25 | 0,07 | 0,16 | 0,14 | 0,18 | 0,19 | 0,19 | 0,16 | 0,11 | 0,07 | 0,06 | 0,04 | 0,07 | 0,13 | 0,06 | 0,08 | 0,16 | 0,18 | 0,08 | 0,00 | 0,04 | 0,16 | 0,02 | 0,13 | 0,11 | 0,08 | 0,05 | 0,09 | 0,08 | 0,04 | 0,02 |
| **A11** | 0,10 | 0,10 | 0,33 | 0,23 | 0,18 | 0,16 | 0,42 | 0,27 | 0,13 | 0,03 | 1,00 | 0,16 | 0,22 | 0,15 | 0,25 | 0,23 | 0,21 | 0,26 | 0,22 | 0,26 | 0,21 | 0,20 | 0,21 | 0,10 | 0,29 | 0,27 | 0,13 | 0,27 | 0,23 | 0,19 | 0,16 | 0,21 | 0,22 | 0,28 | 0,23 | 0,26 | 0,29 | 0,31 | 0,31 | 0,24 | 0,32 | 0,28 |
| **A12** | 0,19 | 0,18 | 0,04 | 0,23 | 0,26 | 0,33 | 0,07 | 0,11 | 0,20 | 0,25 | 0,16 | 1,00 | 0,16 | 0,35 | 0,16 | 0,22 | 0,20 | 0,38 | 0,19 | 0,12 | 0,14 | 0,16 | 0,18 | 0,23 | 0,18 | 0,21 | 0,14 | 0,21 | 0,24 | 0,17 | 0,17 | 0,16 | 0,20 | 0,12 | 0,24 | 0,13 | 0,10 | 0,16 | 0,14 | 0,18 | 0,14 | 0,18 |
| **A13** | 0,21 | 0,22 | 0,11 | 0,24 | 0,34 | 0,16 | 0,34 | 0,05 | 0,20 | 0,07 | 0,22 | 0,16 | 1,00 | 0,27 | 0,21 | 0,16 | 0,18 | 0,22 | 0,17 | 0,16 | 0,11 | 0,16 | 0,21 | 0,25 | 0,16 | 0,25 | 0,10 | 0,20 | 0,16 | 0,18 | 0,15 | 0,23 | 0,19 | 0,19 | 0,17 | 0,18 | 0,25 | 0,24 | 0,22 | 0,20 | 0,17 | 0,26 |
| **A14** | 0,22 | 0,18 | 0,14 | 0,46 | 0,28 | 0,58 | 0,18 | 0,19 | 0,25 | 0,16 | 0,15 | 0,35 | 0,27 | 1,00 | 0,12 | 0,27 | 0,26 | 0,21 | 0,23 | 0,23 | 0,15 | 0,20 | 0,33 | 0,29 | 0,24 | 0,32 | 0,14 | 0,29 | 0,18 | 0,21 | 0,21 | 0,24 | 0,33 | 0,27 | 0,32 | 0,22 | 0,29 | 0,30 | 0,27 | 0,33 | 0,22 | 0,30 |
| **A15** | 0,20 | 0,23 | 0,20 | 0,14 | 0,26 | 0,13 | 0,29 | 0,21 | 0,20 | 0,14 | 0,25 | 0,16 | 0,21 | 0,12 | 1,00 | 0,25 | 0,25 | 0,46 | 0,24 | 0,26 | 0,20 | 0,31 | 0,31 | 0,21 | 0,38 | 0,28 | 0,28 | 0,22 | 0,37 | 0,34 | 0,34 | 0,34 | 0,22 | 0,31 | 0,35 | 0,31 | 0,36 | 0,33 | 0,41 | 0,28 | 0,31 | 0,35 |
| **A16** | 0,24 | 0,22 | 0,25 | 0,32 | 0,27 | 0,28 | 0,18 | 0,24 | 0,20 | 0,18 | 0,23 | 0,22 | 0,16 | 0,27 | 0,25 | 1,00 | 0,31 | 0,24 | 0,33 | 0,28 | 0,20 | 0,24 | 0,24 | 0,23 | 0,26 | 0,24 | 0,21 | 0,29 | 0,24 | 0,22 | 0,12 | 0,20 | 0,33 | 0,25 | 0,31 | 0,21 | 0,28 | 0,31 | 0,28 | 0,29 | 0,26 | 0,26 |
| **A17** | 0,15 | 0,16 | 0,30 | 0,18 | 0,23 | 0,21 | 0,19 | 0,29 | 0,14 | 0,19 | 0,21 | 0,20 | 0,18 | 0,26 | 0,25 | 0,31 | 1,00 | 0,26 | 0,23 | 0,18 | 0,10 | 0,16 | 0,23 | 0,12 | 0,28 | 0,23 | 0,25 | 0,20 | 0,21 | 0,22 | 0,13 | 0,21 | 0,23 | 0,20 | 0,29 | 0,25 | 0,27 | 0,24 | 0,28 | 0,23 | 0,19 | 0,20 |
| **A18** | 0,24 | 0,25 | 0,20 | 0,21 | 0,29 | 0,16 | 0,26 | 0,17 | 0,22 | 0,19 | 0,26 | 0,38 | 0,22 | 0,21 | 0,46 | 0,24 | 0,26 | 1,00 | 0,29 | 0,27 | 0,22 | 0,28 | 0,24 | 0,25 | 0,39 | 0,29 | 0,18 | 0,30 | 0,45 | 0,34 | 0,29 | 0,28 | 0,25 | 0,27 | 0,35 | 0,30 | 0,32 | 0,31 | 0,41 | 0,27 | 0,27 | 0,30 |
| **B1** | 0,22 | 0,22 | 0,26 | 0,23 | 0,27 | 0,21 | 0,27 | 0,23 | 0,24 | 0,16 | 0,22 | 0,19 | 0,17 | 0,23 | 0,24 | 0,33 | 0,23 | 0,29 | 1,00 | 0,51 | 0,45 | 0,42 | 0,35 | 0,27 | 0,38 | 0,37 | 0,17 | 0,58 | 0,37 | 0,36 | 0,29 | 0,33 | 0,35 | 0,37 | 0,39 | 0,28 | 0,37 | 0,38 | 0,37 | 0,34 | 0,33 | 0,35 |
| **B2** | 0,21 | 0,24 | 0,15 | 0,27 | 0,29 | 0,24 | 0,22 | 0,21 | 0,26 | 0,11 | 0,26 | 0,12 | 0,16 | 0,23 | 0,26 | 0,28 | 0,18 | 0,27 | 0,51 | 1,00 | 0,44 | 0,45 | 0,38 | 0,29 | 0,38 | 0,37 | 0,14 | 0,48 | 0,37 | 0,36 | 0,35 | 0,31 | 0,35 | 0,28 | 0,38 | 0,35 | 0,41 | 0,41 | 0,33 | 0,32 | 0,30 | 0,38 |
| **B3** | 0,12 | 0,11 | 0,15 | 0,18 | 0,19 | 0,14 | 0,16 | 0,21 | 0,20 | 0,07 | 0,21 | 0,14 | 0,11 | 0,15 | 0,20 | 0,20 | 0,10 | 0,22 | 0,45 | 0,44 | 1,00 | 0,42 | 0,26 | 0,25 | 0,30 | 0,29 | 0,09 | 0,45 | 0,25 | 0,27 | 0,28 | 0,24 | 0,25 | 0,26 | 0,27 | 0,23 | 0,29 | 0,28 | 0,28 | 0,21 | 0,24 | 0,29 |
| **B4** | 0,23 | 0,22 | 0,23 | 0,24 | 0,24 | 0,16 | 0,28 | 0,22 | 0,22 | 0,06 | 0,20 | 0,16 | 0,16 | 0,20 | 0,31 | 0,24 | 0,16 | 0,28 | 0,42 | 0,45 | 0,42 | 1,00 | 0,49 | 0,41 | 0,45 | 0,43 | 0,18 | 0,49 | 0,33 | 0,45 | 0,41 | 0,43 | 0,32 | 0,39 | 0,39 | 0,40 | 0,42 | 0,41 | 0,46 | 0,33 | 0,34 | 0,45 |
| **B5** | 0,21 | 0,22 | 0,17 | 0,35 | 0,26 | 0,19 | 0,29 | 0,16 | 0,24 | 0,04 | 0,21 | 0,18 | 0,21 | 0,33 | 0,31 | 0,24 | 0,23 | 0,24 | 0,35 | 0,38 | 0,26 | 0,49 | 1,00 | 0,46 | 0,52 | 0,58 | 0,16 | 0,40 | 0,37 | 0,42 | 0,36 | 0,46 | 0,33 | 0,42 | 0,44 | 0,38 | 0,47 | 0,45 | 0,43 | 0,41 | 0,31 | 0,45 |
| **B6** | 0,22 | 0,24 | 0,04 | 0,29 | 0,29 | 0,21 | 0,16 | 0,03 | 0,25 | 0,07 | 0,10 | 0,23 | 0,25 | 0,29 | 0,21 | 0,23 | 0,12 | 0,25 | 0,27 | 0,29 | 0,25 | 0,41 | 0,46 | 1,00 | 0,33 | 0,47 | 0,08 | 0,33 | 0,27 | 0,31 | 0,21 | 0,30 | 0,29 | 0,23 | 0,34 | 0,27 | 0,31 | 0,33 | 0,27 | 0,31 | 0,23 | 0,34 |
| **B7** | 0,24 | 0,20 | 0,29 | 0,25 | 0,29 | 0,21 | 0,37 | 0,25 | 0,26 | 0,13 | 0,29 | 0,18 | 0,16 | 0,24 | 0,38 | 0,26 | 0,28 | 0,39 | 0,38 | 0,38 | 0,30 | 0,45 | 0,52 | 0,33 | 1,00 | 0,59 | 0,37 | 0,44 | 0,50 | 0,55 | 0,48 | 0,48 | 0,33 | 0,48 | 0,49 | 0,51 | 0,53 | 0,49 | 0,64 | 0,34 | 0,43 | 0,46 |
| **B8** | 0,21 | 0,19 | 0,20 | 0,31 | 0,27 | 0,24 | 0,33 | 0,19 | 0,22 | 0,06 | 0,27 | 0,21 | 0,25 | 0,32 | 0,28 | 0,24 | 0,23 | 0,29 | 0,37 | 0,37 | 0,29 | 0,43 | 0,58 | 0,47 | 0,59 | 1,00 | 0,23 | 0,46 | 0,40 | 0,51 | 0,40 | 0,51 | 0,42 | 0,49 | 0,49 | 0,44 | 0,58 | 0,56 | 0,52 | 0,46 | 0,39 | 0,51 |
| **B9** | 0,19 | 0,16 | 0,22 | 0,10 | 0,24 | 0,21 | 0,24 | 0,20 | 0,11 | 0,08 | 0,13 | 0,14 | 0,10 | 0,14 | 0,28 | 0,21 | 0,25 | 0,18 | 0,17 | 0,14 | 0,09 | 0,18 | 0,16 | 0,08 | 0,37 | 0,23 | 1,00 | 0,14 | 0,26 | 0,25 | 0,19 | 0,22 | 0,18 | 0,22 | 0,31 | 0,22 | 0,26 | 0,16 | 0,32 | 0,16 | 0,22 | 0,27 |
| **B10** | 0,20 | 0,21 | 0,19 | 0,26 | 0,24 | 0,26 | 0,27 | 0,23 | 0,24 | 0,16 | 0,27 | 0,21 | 0,20 | 0,29 | 0,22 | 0,29 | 0,20 | 0,30 | 0,58 | 0,48 | 0,45 | 0,49 | 0,40 | 0,33 | 0,44 | 0,46 | 0,14 | 1,00 | 0,37 | 0,43 | 0,35 | 0,40 | 0,36 | 0,38 | 0,43 | 0,37 | 0,46 | 0,42 | 0,42 | 0,37 | 0,36 | 0,39 |
| **B11** | 0,27 | 0,30 | 0,22 | 0,24 | 0,29 | 0,17 | 0,26 | 0,21 | 0,21 | 0,18 | 0,23 | 0,24 | 0,16 | 0,18 | 0,37 | 0,24 | 0,21 | 0,45 | 0,37 | 0,37 | 0,25 | 0,33 | 0,37 | 0,27 | 0,50 | 0,40 | 0,26 | 0,37 | 1,00 | 0,44 | 0,40 | 0,37 | 0,26 | 0,35 | 0,40 | 0,42 | 0,41 | 0,40 | 0,47 | 0,34 | 0,35 | 0,35 |
| **C1** | 0,21 | 0,19 | 0,20 | 0,20 | 0,22 | 0,18 | 0,30 | 0,20 | 0,23 | 0,08 | 0,19 | 0,17 | 0,18 | 0,21 | 0,34 | 0,22 | 0,22 | 0,34 | 0,36 | 0,36 | 0,27 | 0,45 | 0,42 | 0,31 | 0,55 | 0,51 | 0,25 | 0,43 | 0,44 | 1,00 | 0,62 | 0,71 | 0,29 | 0,48 | 0,48 | 0,46 | 0,53 | 0,49 | 0,59 | 0,33 | 0,38 | 0,45 |
| **C2** | 0,22 | 0,23 | 0,13 | 0,18 | 0,24 | 0,18 | 0,28 | 0,17 | 0,20 | 0,00 | 0,16 | 0,17 | 0,15 | 0,21 | 0,34 | 0,12 | 0,13 | 0,29 | 0,29 | 0,35 | 0,28 | 0,41 | 0,36 | 0,21 | 0,48 | 0,40 | 0,19 | 0,35 | 0,40 | 0,62 | 1,00 | 0,66 | 0,26 | 0,48 | 0,43 | 0,40 | 0,49 | 0,45 | 0,53 | 0,29 | 0,41 | 0,45 |
| **C3** | 0,19 | 0,18 | 0,18 | 0,22 | 0,20 | 0,20 | 0,31 | 0,21 | 0,17 | 0,04 | 0,21 | 0,16 | 0,23 | 0,24 | 0,34 | 0,20 | 0,21 | 0,28 | 0,33 | 0,31 | 0,24 | 0,43 | 0,46 | 0,30 | 0,48 | 0,51 | 0,22 | 0,40 | 0,37 | 0,71 | 0,66 | 1,00 | 0,34 | 0,54 | 0,49 | 0,42 | 0,57 | 0,50 | 0,56 | 0,40 | 0,39 | 0,51 |
| **D1** | 0,25 | 0,24 | 0,24 | 0,37 | 0,23 | 0,35 | 0,20 | 0,26 | 0,22 | 0,16 | 0,22 | 0,20 | 0,19 | 0,33 | 0,22 | 0,33 | 0,23 | 0,25 | 0,35 | 0,35 | 0,25 | 0,32 | 0,33 | 0,29 | 0,33 | 0,42 | 0,18 | 0,36 | 0,26 | 0,29 | 0,26 | 0,34 | 1,00 | 0,41 | 0,43 | 0,35 | 0,46 | 0,50 | 0,40 | 0,48 | 0,37 | 0,39 |
| **D2** | 0,11 | 0,10 | 0,29 | 0,22 | 0,15 | 0,18 | 0,38 | 0,25 | 0,11 | 0,02 | 0,28 | 0,12 | 0,19 | 0,27 | 0,31 | 0,25 | 0,20 | 0,27 | 0,37 | 0,28 | 0,26 | 0,39 | 0,42 | 0,23 | 0,48 | 0,49 | 0,22 | 0,38 | 0,35 | 0,48 | 0,48 | 0,54 | 0,41 | 1,00 | 0,45 | 0,45 | 0,61 | 0,58 | 0,71 | 0,48 | 0,55 | 0,55 |
| **D3** | 0,29 | 0,23 | 0,24 | 0,32 | 0,29 | 0,32 | 0,31 | 0,26 | 0,21 | 0,13 | 0,23 | 0,24 | 0,17 | 0,32 | 0,35 | 0,31 | 0,29 | 0,35 | 0,39 | 0,38 | 0,27 | 0,39 | 0,44 | 0,34 | 0,49 | 0,49 | 0,31 | 0,43 | 0,40 | 0,48 | 0,43 | 0,49 | 0,43 | 0,45 | 1,00 | 0,48 | 0,58 | 0,59 | 0,57 | 0,49 | 0,40 | 0,53 |
| **D4** | 0,19 | 0,19 | 0,24 | 0,22 | 0,22 | 0,20 | 0,29 | 0,21 | 0,19 | 0,11 | 0,26 | 0,13 | 0,18 | 0,22 | 0,31 | 0,21 | 0,25 | 0,30 | 0,28 | 0,35 | 0,23 | 0,40 | 0,38 | 0,27 | 0,51 | 0,44 | 0,22 | 0,37 | 0,42 | 0,46 | 0,40 | 0,42 | 0,35 | 0,45 | 0,48 | 1,00 | 0,61 | 0,58 | 0,56 | 0,43 | 0,41 | 0,43 |
| **D5** | 0,23 | 0,20 | 0,28 | 0,31 | 0,23 | 0,25 | 0,39 | 0,24 | 0,15 | 0,08 | 0,29 | 0,10 | 0,25 | 0,29 | 0,36 | 0,28 | 0,27 | 0,32 | 0,37 | 0,41 | 0,29 | 0,42 | 0,47 | 0,31 | 0,53 | 0,58 | 0,26 | 0,46 | 0,41 | 0,53 | 0,49 | 0,57 | 0,46 | 0,61 | 0,58 | 0,61 | 1,00 | 0,77 | 0,68 | 0,61 | 0,47 | 0,57 |
| **D6** | 0,25 | 0,23 | 0,30 | 0,36 | 0,25 | 0,26 | 0,34 | 0,24 | 0,18 | 0,05 | 0,31 | 0,16 | 0,24 | 0,30 | 0,33 | 0,31 | 0,24 | 0,31 | 0,38 | 0,41 | 0,28 | 0,41 | 0,45 | 0,33 | 0,49 | 0,56 | 0,16 | 0,42 | 0,40 | 0,49 | 0,45 | 0,50 | 0,50 | 0,58 | 0,59 | 0,58 | 0,77 | 1,00 | 0,65 | 0,66 | 0,44 | 0,54 |
| **D7** | 0,20 | 0,16 | 0,33 | 0,24 | 0,24 | 0,20 | 0,40 | 0,28 | 0,18 | 0,09 | 0,31 | 0,14 | 0,22 | 0,27 | 0,41 | 0,28 | 0,28 | 0,41 | 0,37 | 0,33 | 0,28 | 0,46 | 0,43 | 0,27 | 0,64 | 0,52 | 0,32 | 0,42 | 0,47 | 0,59 | 0,53 | 0,56 | 0,40 | 0,71 | 0,57 | 0,56 | 0,68 | 0,65 | 1,00 | 0,52 | 0,56 | 0,59 |
| **D8** | 0,19 | 0,19 | 0,27 | 0,36 | 0,22 | 0,27 | 0,28 | 0,24 | 0,17 | 0,08 | 0,24 | 0,18 | 0,20 | 0,33 | 0,28 | 0,29 | 0,23 | 0,27 | 0,34 | 0,32 | 0,21 | 0,33 | 0,41 | 0,31 | 0,34 | 0,46 | 0,16 | 0,37 | 0,34 | 0,33 | 0,29 | 0,40 | 0,48 | 0,48 | 0,49 | 0,43 | 0,61 | 0,66 | 0,52 | 1,00 | 0,40 | 0,46 |
| **D9** | 0,16 | 0,14 | 0,33 | 0,24 | 0,19 | 0,22 | 0,31 | 0,28 | 0,14 | 0,04 | 0,32 | 0,14 | 0,17 | 0,22 | 0,31 | 0,26 | 0,19 | 0,27 | 0,33 | 0,30 | 0,24 | 0,34 | 0,31 | 0,23 | 0,43 | 0,39 | 0,22 | 0,36 | 0,35 | 0,38 | 0,41 | 0,39 | 0,37 | 0,55 | 0,40 | 0,41 | 0,47 | 0,44 | 0,56 | 0,40 | 1,00 | 0,62 |
| **D10** | 0,16 | 0,13 | 0,25 | 0,32 | 0,22 | 0,27 | 0,37 | 0,23 | 0,16 | 0,02 | 0,28 | 0,18 | 0,26 | 0,30 | 0,35 | 0,26 | 0,20 | 0,30 | 0,35 | 0,38 | 0,29 | 0,45 | 0,45 | 0,34 | 0,46 | 0,51 | 0,27 | 0,39 | 0,35 | 0,45 | 0,45 | 0,51 | 0,39 | 0,55 | 0,53 | 0,43 | 0,57 | 0,54 | 0,59 | 0,46 | 0,62 | 1,00 |
