# Supplementary figures and images for: Transcultural adaptation and psychometric study of the French version of the nursing home survey on patient safety culture questionnaire
Source: BMC Health Serv Res. 2019 Jul 15;19:490. doi: 10.1186/s12913-019-4333-5 (PMC6631961; doi:10.1186/s12913-019-4333-5)

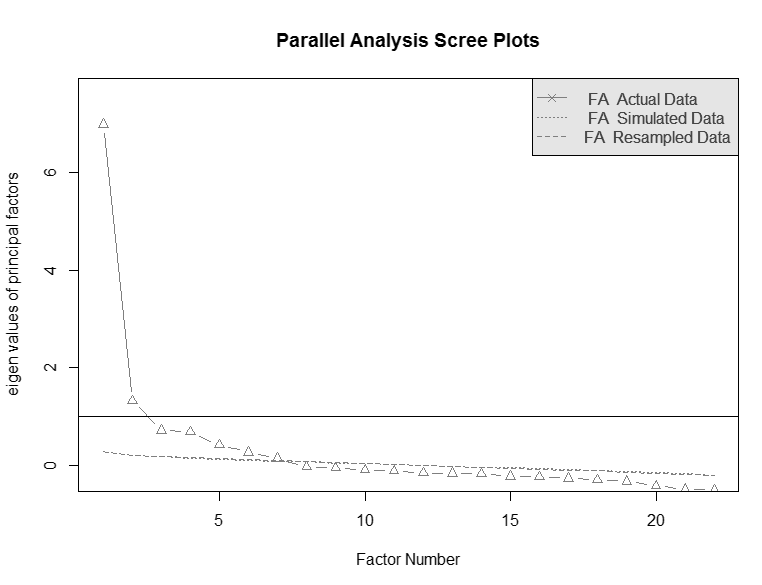

Supplement: Supplementary file 5 — Parallel analysis scree plots. (PNG 12 kb) [file 12913_2019_4333_MOESM5_ESM.png]

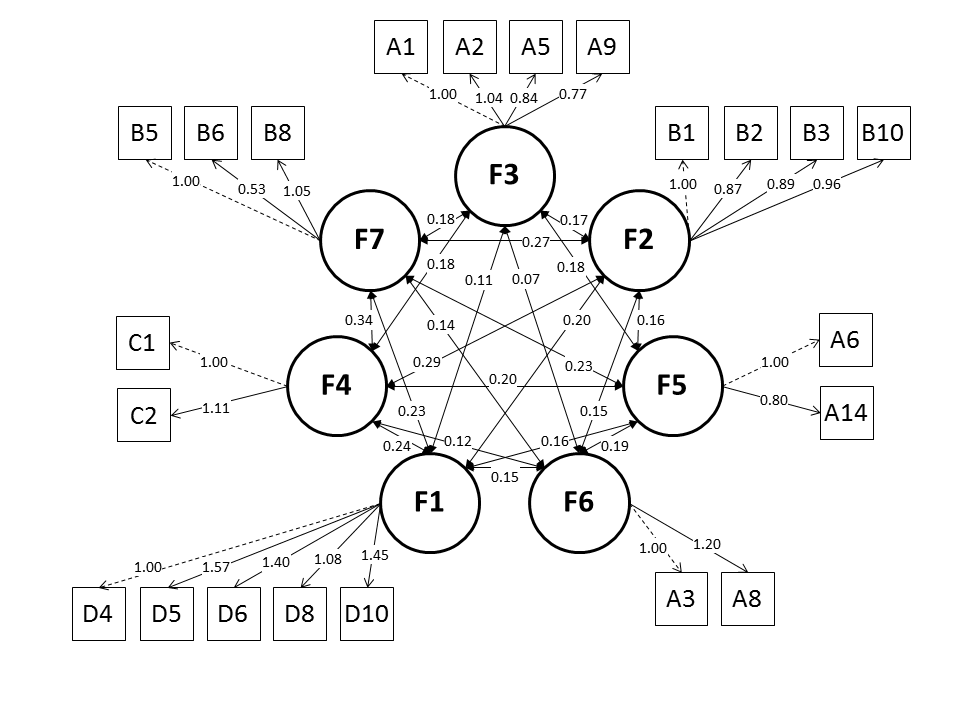

Supplement: Supplementary file 8 — Structural Equation Model diagram. (PNG 28 kb) [file 12913_2019_4333_MOESM8_ESM.png]
