# Supplementary material for: Transcultural adaptation and psychometric study of the French version of the nursing home survey on patient safety culture questionnaire
Source: BMC Health Serv Res. 2019 Jul 15;19:490. doi: 10.1186/s12913-019-4333-5 (PMC6631961; doi:10.1186/s12913-019-4333-5)
Supplement: Supplementary file 6 — Description of item responses per answer choice (percentages) . (PDF 171 kb) [file 12913_2019_4333_MOESM6_ESM.pdf]

Additional file 6

Table: Description of item responses per answer choices (percentages)

Dimensions / Items	Strongly disagree	Disagree	Neither agree nor disagree	Agree	Strongly agree	Missing value
Teamwork						
A1 – Staff in the nursing home treat each other with respect	1.5	4.5	35.1	44.8	14.1	2.7
A2 – Staff support one another in this nursing home	1.7	5.3	43.0	38.9	11.1	3.6
A5 – Staff feel like they are part of a team	1.7	4.5	24.5	51.9	17.4	5.4
A9 – When someone gets really busy in this nursing home, other staff help out	1.8	4.9	37.8	41.7	13.8	6.1
Staffing						
A3 –We have enough staff to handle the workload	25.7	29.3	30.3	12.3	2.3	4.2
A8R – Staff have to hurry because they have too much work to do	2.1	8.3	23.3	39.3	26.9	5.7
Compliance with procedures						
A6R – Staff use shortcuts to get their work done faster	11.0	34.0	30.7	21.0	3.3	11.9
A14R – To make work easier, staff often ignore procedures	12.5	50.8	26.3	9.3	1.1	12.0
Supervisor expectations and actions promoting resident safety						
C1 – My supervisor listens to staff ideas and suggestions about resident safety	1.0	3.9	14.0	45.9	35.2	9.0
C2 – My supervisor says a good word to staff who follow the right procedures	4.3	9.4	12.4	35.0	38.9	13.2
Overall perceptions of resident safety / Organizational learning						
D6 – This nursing home does a good job keeping resident safe	0.5	3.1	14.7	55.7	26.0	5.9
D8 – This nursing home is a safe place for residents	0.4	1.9	8.8	56.6	32.2	5.5
D4 – It is easy to make changes to improve resident safety in this nursing home	1.4	8.3	26.5	52.3	11.4	12.6
D5 – This nursing home is always doing things to improve resident safety	0.7	4.2	17.2	50.3	27.5	8.5
D10 – When this nursing home makes changes to improve resident safety, it checks to see if the changes worked	3.0	9.6	19.0	43.5	24.9	19.1

Dimensions / Items	Never	Rarely	Sometimes	Most of the time	Always	Missing value
Handoffs (transfer of information)						
B1 – Staff are told what they need to know before taking	0.8	5.4	16.6	56.7	20.4	10.0

care of a resident for the first time						
B2 – Staff are told right away when there is a change in a resident's care plan	0.6	3.6	16.4	57.4	21.9	9.6
B3 – We have all the information we need when residents are transferred from the hospitals	1.0	8.8	27.2	49.7	13.3	10.2
B10 – Staff are given all the information they need to care for residents	0.5	2.5	13.6	62.3	21.2	8.5
Feedback and communication about incidents						
B5 – In this nursing home, we talk about ways to keep incidents from happening again	0.9	4.5	14.2	49.6	30.7	7.8
B6 – Staff tell someone if they see something that might harm a resident	0.2	0.9	6.7	48.0	44.2	6.6
B8 – In this nursing home, we discuss ways to keep residents safe from harm	0.7	3.6	14.3	46.0	35.3	7.2

NOTE: items flagged R: response scores are reversed to preserve the negative meaning
