# Supplementary material for: Transcultural adaptation and psychometric study of the French version of the nursing home survey on patient safety culture questionnaire
Source: BMC Health Serv Res. 2019 Jul 15;19:490. doi: 10.1186/s12913-019-4333-5 (PMC6631961; doi:10.1186/s12913-019-4333-5)
Supplement: Supplementary file 7 — Structural Equation Model outputs. (PDF 237 kb) [file 12913_2019_4333_MOESM7_ESM.pdf]

Additional file 7

Table 1: Indicators to assess the global fit of the final structural equation model (R output)

npar	fmin	chisq	df	pvalue	baseline.chisq
65.000	0.435	346.275	188.000	0.000	4165.487
baseline.df	baseline.pvalue	cfi	tli	nnfi	rfi
231.000	0.000	0.960	0.951	0.951	0.898
nfi	pnfi	ifi	rni	logl	unrestricted.logl
0.917	0.746	0.960	0.960	-9101.495	-8927.923
aic	bic	ntotal	bic2	rmsea	rmsea.ci.lower
18332.991	18592.273	399.000	18386.025	0.046	0.038
rmsea.ci.upper	rmsea.pvalue	rmr	rmr_nomean	srmr	srmr_bentler
0.054	0.803	0.031	0.031	0.044	0.044
srmr_bentler_nomean	srmr_bollen	srmr_bollen_nomean	srmr_mplus	srmr_mplus_nomean	cn_05
0.044	0.044	0.044	0.044	0.044	255.001
cn_01	gfi	agfi	pgfi	mfi	ecvi
272.284	0.928	0.903	0.689	0.820	1.197

Table 2: Parameter estimates of the final structural equation model (R output)

lhs	op	rhs	Est	se	z	pvalue
f1	=~	A1	1.000	0.000	NA	*NA*
f1	=~	A2	1.042	0.063	16.541	*0.000*
f1	=~	A5	0.842	0.058	14.556	*0.000*
f1	=~	A9	0.772	0.065	11.911	*0.000*
f2	=~	B1	1.000	0.000	NA	*NA*
f2	=~	B2	0.867	0.066	13.206	*0.000*
f2	=~	B3	0.894	0.075	11.886	*0.000*
f2	=~	B10	0.962	0.064	15.005	*0.000*
f3	=~	B5	1.000	0.000	NA	*NA*
f3	=~	B6	0.530	0.056	9.411	*0.000*
f3	=~	B8	1.053	0.073	14.471	*0.000*
f4	=~	C1	1.000	0.000	NA	*NA*
f4	=~	C2	1.105	0.085	13.050	*0.000*
f5	=~	D4	1.000	0.000	NA	*NA*
f5	=~	D5	1.567	0.116	13.459	*0.000*
f5	=~	D6	1.395	0.104	13.364	*0.000*
f5	=~	D8	1.081	0.092	11.763	*0.000*
f5	=~	D10	1.446	0.126	11.501	*0.000*
f6	=~	A6	1.000	0.000	NA	*NA*
f6	=~	A14	0.795	0.095	8.379	*0.000*
f7	=~	A3	1.000	0.000	NA	*NA*
f7	=~	A8	1.199	0.158	7.567	*0.000*
A1	~~	A1	0.251	0.026	9.555	*0.000*
A2	~~	A2	0.209	0.025	8.294	*0.000*

A5	~~	A5	0.291	0.025	11.421	*0.000*
A9	~~	A9	0.467	0.037	12.739	*0.000*
B1	~~	B1	0.304	0.028	10.890	*0.000*
B2	~~	B2	0.303	0.026	11.703	*0.000*
B3	~~	B3	0.470	0.038	12.475	*0.000*
B10	~~	B10	0.192	0.020	9.401	*0.000*
B5	~~	B5	0.320	0.029	10.955	*0.000*
B6	~~	B6	0.301	0.023	13.210	*0.000*
B8	~~	B8	0.224	0.025	8.883	*0.000*
C1	~~	C1	0.183	0.033	5.550	*0.000*
C2	~~	C2	0.599	0.056	10.639	*0.000*
D4	~~	D4	0.436	0.032	13.432	*0.000*
D5	~~	D5	0.144	0.016	8.828	*0.000*
D6	~~	D6	0.130	0.014	9.442	*0.000*
D8	~~	D8	0.256	0.020	12.752	*0.000*
D10	~~	D10	0.522	0.040	12.922	*0.000*
A6	~~	A6	0.437	0.066	6.601	*0.000*
A14	~~	A14	0.283	0.042	6.717	*0.000*
A3	~~	A3	0.488	0.070	6.928	*0.000*
A8	~~	A8	0.292	0.091	3.224	*0.001*
f1	~~	f1	0.459	0.051	9.023	*0.000*
f2	~~	f2	0.395	0.048	8.221	*0.000*
f3	~~	f3	0.379	0.048	7.936	*0.000*
f4	~~	f4	0.517	0.057	9.120	*0.000*
f5	~~	f5	0.249	0.039	6.432	*0.000*
f6	~~	f6	0.539	0.085	6.325	*0.000*
f7	~~	f7	0.501	0.087	5.793	*0.000*
f1	~~	f2	0.169	0.028	5.959	*0.000*
f1	~~	f3	0.179	0.029	6.171	*0.000*
f1	~~	f4	0.184	0.032	5.755	*0.000*
f1	~~	f5	0.112	0.022	5.163	*0.000*
f1	~~	f6	0.180	0.036	5.072	*0.000*
f1	~~	f7	0.069	0.030	2.260	*0.024*
f2	~~	f3	0.272	0.032	8.395	*0.000*
f2	~~	f4	0.289	0.034	8.421	*0.000*
f2	~~	f5	0.204	0.026	7.721	*0.000*
f2	~~	f6	0.160	0.033	4.797	*0.000*
f2	~~	f7	0.147	0.032	4.527	*0.000*
f3	~~	f4	0.335	0.037	9.139	*0.000*
f3	~~	f5	0.234	0.029	8.148	*0.000*
f3	~~	f6	0.227	0.037	6.205	*0.000*
f3	~~	f7	0.135	0.032	4.198	*0.000*
f4	~~	f5	0.241	0.030	8.126	*0.000*
f4	~~	f6	0.200	0.039	5.164	*0.000*
f4	~~	f7	0.122	0.035	3.489	*0.000*
f5	~~	f6	0.158	0.028	5.642	*0.000*

f5	~~	f7	0.145	0.028	5.222	*0.000*
f6	~~	f7	0.189	0.042	4.514	*0.000*
